# Using Open, Public Data for Security Provision: Ethical Perspectives on Risk-Based Border Checks in the EU

**DOI:** 10.1007/s41125-023-00092-4

**Published:** 2023-06-01

**Authors:** Sebastian Weydner-Volkmann

**Affiliations:** Ruhr University Bochum, Bochum, Germany

**Keywords:** Border checks, Open-source intelligence, Security production, Risk-based checks, EU, Surveillance

## Abstract

This article explores the use of open-source intelligence (OSINT) techniques as part of data-driven border checks in the EU. While the idea to group travelers into risk categories in order to differentiate the intensity of border checks has been criticized for its likely impact on privacy and other fundamental rights, the exclusive use of “open,” “public” data was proposed as an alternative that mitigates these issues. However, OSINT remains a rather vague term, as it is unclear what constitutes “open” or “public” data, how the use of such techniques would contribute to the production of security, and whether its use actually mitigates most ethical issues. The goal of this article is to contribute toward a situated answer to these questions. It will provide groundwork by clarifying what OSINT practices could entail in the context of the European border checks regime and by developing an ethical perspective on these practices. I will show that the impact depends not so much on the public availability of the analyzed data, but on the specifics of the implementation of OSINT techniques. Thus, certain uses of OSINT continue to raise severe privacy and fundamental rights issues.

## Introduction

The provision of public security in the EU has grown increasingly dependent on the use of data.^1^ This is especially apparent in the shift in law enforcement toward proactive or preemptive measures (Leese 2014, p. 495; Orrù 2021, p. 300). Some of these measures presume that an existential threat to public security exists, but that law enforcement agents do not yet know *who or what exactly* may pose this threat. In order to deal with this issue, agents may *construct suspicion* through the collection and processing of data (Zurawski 2015, pp. 34–37; Salter 2008, pp. 254–55): In such cases, law enforcement agents assume that the analysis of potentially relevant data can be used to define information patterns that allow for assigning *risks* to individuals or smaller groups, i.e., likelihoods of posing a threat to public security. This security practice has been termed “social sorting” in the international surveillance literature (Lyon 2006; Adey 2006).

For such measures, the steps of data collection and analysis *precede* the construction of suspicion. Therefore, it is unknown before the collection, what data should be included, and what data are likely to be irrelevant. From a law enforcement perspective, these data-driven measures, therefore, require rather broad collections of mostly unstructured data, which, in turn, demands the use of “big data” applications for analysis (Zurawski 2015, p. 33).

Once information patterns and risk categories are defined, they can be used, for example, to differentiate security checks in airports or at border crossing points (Lyon 2006, p. 404). Here, the intensity of checks is meant to be differentiated based on a risk classification. The higher the risk assigned to a traveler, the more intense the security checks—a concept that has been termed “risk-based screening” (Weydner-Volkmann 2017, pp. 60–62). However, as the literature highlights, data-driven risk assessment keeps conflicting with fundamental values (Adey 2006, p. 196; Leese 2014, pp. 506–507; Weydner-Volkmann 2017, pp. 62–76; Orrù 2021, p. 230; Rademacher 2017, p. 416). After all, the large-scale collection and analysis of personal data are prone to conflict with the rights to private life and the protection of personal data (Office for Democratic Institutions and Human Rights 2021, pp. 8–11).

This, among other things, has led to the idea of avoiding personal data and of using publicly available, “open” data instead. Where only public information is used for risk assessment, it seems reasonable to assume that privacy and data protection issues will not apply. As Hälterlein (2020, pp. 50–51) writes, police agencies have started to increasingly make use of corresponding “open source” intelligence (OSINT) techniques. OSINT remains a rather vague term, however, as it is unclear what constitutes “open” or “public” data, how the use of such techniques would contribute to, for example, border checks, and whether its use actually mitigates ethical issues.

The goal of this article is to contribute toward a situated answer to these questions. It will provide groundwork by clarifying what OSINT practices could entail in the context of the border checks regime that governs the Schengen Area and by developing an ethical perspective on these practices. I will show that the ethical impact depends not so much on the public availability of the analyzed data, but on the specifics of the implementation of OSINT techniques. As we will see, certain uses of OSINT will still constitute what Nissenbaum (2004, p. 119) has termed violations of contextual integrity and other ethical issues.

For this, I will begin by establishing the context of data-driven border checks, first by sketching relevant historical developments (Sect.  2.1) then by introducing the “logic” of border checks within the EU’s border management regime for the Schengen Area (Sect.  2.2). This will provide the basis to then discuss from an ethical perspective how data-driven, risk-based border checks come into conflict with other values, such as privacy and data protection (Sect. 3). I will then specify what role OSINT can play for two main variants of risk classification (Sects. 4.1 and 4.3), and I will show for each of them how different implementations of OSINT may mitigate or exacerbate privacy, data protection, and related ethical issues (Sects. 4.2 and 4.4).

## Border Checks in Context

### Data-Driven Border Checks

The abolishment of checks at national borders in the 1985 Schengen Agreement was seen as a major challenge to the provision of public security. Hence, “complementary measures to safeguard internal security” were introduced as a counterbalance (EC 2000, Agreement Art 17; cf. Goetz 2018, p. 228). As Goetz (2018, pp. 229–330) argues, extensive exchange of information became a central part of the EU’s *integrated border management* strategy and its vision for implementation (*smart borders package*). The processing and sharing of what is deemed security relevant information mostly took form in database systems such as the Schengen Information System (SIS, SIS II), the Visa Information System (VIS), as well as Eurodac (Goetz 2018, p. 245; Orrù 2021, p. 165). Key actors in the EU see such digital measures in border control in the Schengen Area as “an important key element to maintain and to enhance the internal security of the European Union” (Council of the EU 2015, p. 7; cf. Goetz 2018, p. 230; Orrù 2021, pp. 171–72). As Leese (2016, p. 416) shows, however, economic factors were also highly relevant for the EU’s plans of a registered travelers program.^2^

In regard to the original *collection* of security relevant information, the EU’s Passenger Name Record (PNR) Directive (EU 2016b) was long set to become a cornerstone for a proactive, risk-based approach to public security provision. Originally proposed in 2007, it conceptualizes the

“use of passenger data as a means to create new criteria for the identification of terrorist and transnational criminals … PNR data were considered a major factor in providing much-needed information for fighting terrorism and serious crime, as well as for border control and migration issues” (Leese 2014, p. 495).

PNR data are collected as part of the commercial processes in civil aviation; they contain traveler information such as name, date of birth, address, etc., but also information regarding the booking and billing process, accompanying travelers, as well as any available information on the complete travel itinerary (EU 2016b, Art. 12).

As mentioned earlier, the fundamental rights issues of a proactive, risk-based mode of security provision have received considerable attention. In line with an earlier opinion formulated by the court (ECJ 2017, esp. paragraphs 124 and 126), a recent ruling of the European Court of Justice on the use and communication of PNR data has largely curtailed practices of using personal data to construct suspicion and demanded safeguards against false positives and discriminating biases (ECJ 2022, esp. rulings 5.–8.). Member states’ implementations of the EU’s PNR directive will, therefore, have to be revised. While this shows that the use of personal data in risk-based border checks raises severe ethical, political, and legal issues, it also shows that the construction of information patterns for traveler risk classification *creates a demand* for ever more personal data (Salter 2008, p. 255; Weydner-Volkmann 2017, p. 72).

On the other hand, the exclusive use of public information for risk assessment seems like a viable way out; as mentioned above, police agencies have started to make use of such open-source intelligence (OSINT) techniques. As Hälterlein (2020, pp. 50–51) underscores, the analysis of openly available, public data is often seen as a *technological* challenge that can be met with machine learning methods. Similarly, Leese (2016, p. 421) highlights the Smart Borders Package as an example for a “turn to technology as a viable solution for security problems.” Sections 4.1 and 4.3 will discuss the role of OSINT in border checks in more detail by discussing concrete examples from a European Horizon 2020 research project. In the next section, I will establish an understanding of how border checks work in the context of the EU’s legal framework.

### The Logic of Border Checks

The Schengen Borders Code (EU 2016a, Art. 2) defines “border checks” as one of the two main activities that make up “border control”: in contrast with “border surveillance,” which deals with enforcing that borders are only crossed at “border crossing points” (BCPs), “border checks” refers to “the checks carried out at border crossing points, to ensure that persons, including their means of transport and the objects in their possession, may be authorised to enter the territory of the Member States or authorised to leave it” (EU 2016a, Art. 2). By including objects in the possession of persons, border checks touch on the customs regime that governs the movement of *goods* across borders. As such, those two regimes each follow their own distinct set of goals. I will focus on *border checks*, only.

Border checks essentially provide two main functions: (1) They perform access and egress control in a spatial, territorial sense based on (2) information revealed by inspection. In line with an earlier conceptualization in aviation security (Weydner-Volkmann 2018, pp. 127–28), I will call the first function “access and egress control function” and the second “revelatory function.” Access and egress control presupposes the idea of a spatial separation. The act of “crossing over” is subject to certain regulatory conditions being met; otherwise, it is denied.

If access and egress control is to be performed in a meaningful way, it must be able to deal with situations where travelers deliberately make false claims. Hence, it is dependent on the revelatory function, which needs to (a) reveal the identity of persons and their belongings to border guards or customs officers. This is so that they can be subsumed under certain regulatory categories, which, in turn, imply different conditions under which they can or cannot move across the border (e.g., “persons enjoying the right of free movement under Union law” or “goods made from endangered and protected animals”). Furthermore, the revelatory function needs to (b) uncover whether a person’s identity has been deliberately falsified or the presence of certain goods or other persons concealed from plain view. In doing so, border guards not only inspect persons, documents, and goods, but they also collect data (e.g., for entries in the EU’s planned Entry-Exit-System, EES), and they make use of previously collected and processed information (e.g., by checking databases such as SIS, VIS, Eurodac, or, prospectively, OSINT data).

Within the Schengen Area, border checks at border crossing points are only foreseen at the EU’s external border, not at internal borders (EU 2016a, Art. 22)—although temporary border checks are allowed in certain exceptional situations such as during the COVID-19 pandemic (EU 2016a, Art. 25). Similarly, certain types of police checks that do “not have an effect equivalent to border checks” (EU 2016a, Art. 23) may be performed. For the purpose of this paper, border checks can thus be conceptualized as *performing the access and egress control function at the external borders of the Schengen Area with regard to the movement of persons (and goods brought along) based on the outcome of the revelatory function* (Weydner-Volkmann 2020)*.*

The purposes of performing those two functions are governed by separate regimes that operate in different normative contexts. One example would be preventing migrants entering a country who do not have “sufficient means of subsistence” [EU 2016a, Art. 6 (1) c], another would be preventing perpetrators of serious crime such as terrorism from entering or leaving a country. There is, hence, no singular and consistently specifiable end with regard to border checks. In the abstract, the purpose of performing the two main functions of border checks can only be defined as *allowing the governance of persons who cross the border by enforcing the corresponding regimes*.

While for the third-country nationals, “thorough checks” are mandatory upon entry [EU 2016a, Art. 8 (3) a vi] and “whenever possible” on exit [EU 2016a, Art. 8 (3) g iii], EU nationals and persons enjoying the right of free movement under Union law may or may not be subject to threat-related measures:

“… on a non-systematic basis … border guards may consult national and European databases in order to ensure that such persons do not represent a genuine, present and sufficiently serious threat to the internal security, public policy, international relations of the Member States or a threat to the public health … The consequences of such consultations shall not jeopardise the right of entry of persons enjoying the right of free movement under Union law into the territory of the Member State concerned …” (EU 2016a, Art. 8 (2)).

Of the four threats explicitly mentioned, only the threat to public health is specified further in the definitory section (EU 2016a, Art. 2). As Leese (2016, 425) notes, the other threats remain strikingly unspecific, which “relocates the decision authority back to the member states.” More recent amendments to the Schengen Border Code introduced *risk profiling* explicitly as a basis for border checks as part of ETIAS (EU 2018, Art. 33). Here, three broader categories were used: risks of illegal migration and visa “overstays,” epidemic risks, and security risks. Again, security risks are unspecifically defined as “risk of a threat to public policy, internal security and international relations” (EU 2018, Art. 3). While each of these broader categories warrants further examination, OSINT techniques are predominantly discussed in the context of threats to internal security. I will, therefore, focus the discussion on these types of security risks.

Depending on the specific measures used during border checks, further EU legislation may be involved and, through that, further specification of the purposes pertinent to those measures may be specified. For example, the measure of PNR data processing is bound to the purpose of fighting terrorist offenses and serious crime [EU 2016b, Art. 1 (2)], but not allowed for the purposes of assessing a person’s means of subsistence and the risk of overstaying their visa. At the moment, however, there is no legislation governing the use of OSINT as part of border checks. Therefore, its role cannot be deduced from the legal framework. Instead, Sects . 4.1 and 4.3 will discuss potential roles of OSINT. In the next section, an ethical perspective is introduced on how data-driven, risk-based checks at the Union’s external borders may conflict with other values, such as the privacy.

## Value Conflicts in Data-Driven Border Checks

The last section outlined the logic that governs the EU’s border checks regime for the Schengen Area. Since, currently, the regime provides no clear basis for using “open, public data” for risk classification of travelers, what will and will not be legally permissible is still in flux and will depend on national and European legislative processes. An ethical analysis such as the one presented here may be considered relevant input to these processes. For this, I will refer to established value concepts (such as privacy and non-discrimination) not in *purely legal* terms or in terms of data protection *legislation*, but as concepts that are part of a broader philosophical and societal discussion.^3^

In order to attain their purpose, border checks always have to reveal information about persons or their belongings that are not readily and publicly available or divulged voluntarily—i.e., information that is private. Hence, border checks necessarily come into conflict with privacy-related values. Unfortunately, the concept of privacy is notoriously hard to define and has rightly been labeled “a concept in disarray. Nobody can articulate what it means” (Solove 2009, p. 1). Due to this, Solove (2009, p. 40) treats “privacy” as a cluster of issues united by what Wittgenstein would call “family resemblance.”

One prominent trait running through much of the family of those “privacy aspects” is the idea of information that commonly enjoys protection and shielding from others, so as to allow for respect and free development of an individual’s personality. As Nissenbaum (2004, p. 124) has argued in specific regard of surveillance activities, the integrity of informational privacy is not so much dependent on *properties of the respective data* but on *contextual norms*.

“A central tenet of contextual integrity is that there are no arenas of life not governed by norms of information flow, no information or spheres of life for which ‘anything goes.’ Almost everything—things that we do, events that occur, transactions that take place—happens in a context not only of place but of politics, convention, and cultural expectation” (Nissenbaum 2004, p. 137).

While there are many other conceptions of privacy one could use, highlighting flows of data in normatively relevant contexts helps identifying the potential privacy impact of OSINT. I will, therefore, loosely draw from Nissenbaum’s idea of contextual integrity in my analysis of the privacy implications of OSINT techniques in Sect. 4. Instead of applying her larger framework, however, I will situate OSINT techniques as part of two main variants of risk-based border checks and then develop a broader ethical analysis by building on the logic of border checks outlined in Sect. 2.2.

First, however, one must note that claims to informational privacy are often understood almost synonymous to claims to data protection. As Poscher (2017, p. 131) notes, however, “Europe is still not at ease with the right to data protection … Fundamental rights usually protect a general or specific liberty or equality interest. But what, specifically, should that be in the case of the right to data protection?” In answer to a wave of criticism leveled in this vein against the right, he proposes that data protection should not be considered a right on its own at all, but rather as a “systematic enhancement of other fundamental rights” (Poscher 2017, p. 136). Data protection, he argues, is essentially about *abstract dangers*:

“The collection of data as such does no harm … It is only the use of data in certain contexts that might cause a violation of liberty or equality interests. The collection of personal data about political or religious convictions … is generally prohibited, for example, because of the potential that it could be misused by the state to discriminate against certain political or religious groups … It does not require concrete evidence that misuse of the data has taken place, or even that such a misuse is about to occur” (Poscher 2017, p. 137).

Consequently, he argues that, when dealing with issues of data protection, we cannot limit ourselves to privacy rights or the specifics of data protection regulation, but rather spell out to what extent a certain form of data collection and processing is posing an abstract danger for the exercise of what kind of fundamental right (Poscher 2017, p. 137). I will follow Poscher in this clarification as part of my broader ethical analysis; hence, I will assume that OSINT may collide with further fundamental value concepts besides privacy.^4^

Here, the potential for unfair or discriminatory distributions of impact is highly relevant, as the intensity of ensuing checks for travelers deemed “high risk” is likely to be considerably higher than for other travelers in risk-based border checks. As Boshammer (2008, p. 232) notes, discrimination inherently entails a comparative element. Lippert-Rasmussen (cit. by Altman 2015) explicates this further:

“Unlike other *prima facie* morally wrong acts, such as lying, hurting, or manipulating, one cannot discriminate against some unless there are others who receive (or who would receive) better treatment at one’s hands … I can rebut an accusation of having discriminated against someone by saying that I would have treated anyone else at least as badly in that situation.”

However, in the academic discussion of discrimination, it has been noted that not just any differential treatment of groups is to be seen as discrimination in the thick normative sense of *illegitimate* differentiation—and not just any logically conceivable group of persons is to seen as relevant here (Altman 2015). In order to understand when differential treatment is illegitimate, Altman (2015) refers to the concept of “salient social groups”: It denotes specific groups that are, for historic or cultural reasons, commonly considered to be vulnerable or put at a structural societal disadvantage. As Boshammer (2008, p. 233) notes, legal prohibitions of discrimination originally referenced skin color, sex, ethnicity, “race,” and religious convictions; more recently, however, many other categories have been added such as age, sexual orientation, and social or health status.

Further abstract dangers to be considered relate to the transparency of OSINT and the connected issue of a potential lack of accountability when opaque techniques obstruct travelers in seeking legal redress. Another danger consists in societal chill, i.e., the potential that security provision results in people ceasing *legitimate* or even *democratically desired* activities (Solove 2009, p. 178). In the following, Sects.  4.1 and 4.3 will discuss the potential role of OSINT as part of two main variants of risk-based border checks. Sections  4.2 and 4.4 will then apply the value concepts outlined above. Reminiscent of Koops et al.’ (2013) contribution on *legal* compliance, these sections may be considered an *ethical* contribution to OSINT practices that consider privacy and ethics “by design.”

## The Use of Open, Public Data in Risk-Based Border Checks

In this section, I will specify how “open” or “public” data may be used as part of data-driven risk classification in border checks and show that it is not so much the public availability of the analyzed data, but rather the specifics of the implementation that is decisive for OSINT’s ethical impact. I will start from a conceptual differentiation of risk assessment methods suitable for risk-based border checks and discuss OSINT in a situationally embedded fashion. For this, I will build on the logic of border checks outlined in Sect. 2.2 and refer to examples from the applied project TRESSPASS^5^ on risk-based border checks.

As has been shown for aviation security (Weydner-Volkmann 2017, pp. 62–76), it is helpful from an ethics perspective to distinguish three main variants of risk classification based on the kind of information that is used: Either (1) only purely situational data are used; or traveler-related data are used. In the latter case, these traveler data can either (2) refer to additional “background information” on travelers, or (3) to the travelers’ behavior during the checks and at the border crossing point. Since behavior analysis techniques are commonly not considered to be part of OSINT, only the first two variants are relevant for this article.

The collection and use of “background information” on travelers for risk profiling can only be considered OSINT, if the information is “openly” and “publicly” available. As Ganguly (2022, p. 24) writes, one of the earliest definitions of OSINT can be traced to former CIA officer Robert Steele:

“By Open Source we refer to publicly available information appearing in print or electronic form … It may be disseminated to a broad public, as are the mass media, or to a more select audience … Whatever form it takes, Open Source involves no information that is: classified at its origin; is subject to proprietary constraints (other than copyright); is the product of sensitive contacts with the U.S.” (Steele 1995, p. 457; cit. by Ganguly 2022, p. 24).

Following this definition, OSINT data used for risk profiling could relate to information that has been made public by travelers themselves (e.g., by posting publicly on social media) or that was made public by third parties. This could involve journalistic sources (e.g., for persons of public interest), but it could also involve social media and blog postings of travelers’ friends. While access to sources of good, open-source information was seen as a challenge in the early phases of OSINT, the advent of the internet, social media, and other digital technologies has led to a different type of challenge: “to sift through the millions of sources publicly available and find the right piece of evidence … Most open-source investigations are now conducted exclusively online” (Ganguly 2022, pp. 25–26).

### Surface and Deep Web OSINT for Situational Risk Assessment

For situational risk-based border checks, the revelatory function makes use of *contextual* or *situational* risk information, not on information related to individual travelers. An example for this could be a situation, in which a large amount of plastic explosives has been stolen in a neighboring third country. As part of risk-based border checks, one may assume that this leads to a heightened risk that plastic explosives are smuggled across the EU’s external border into the Schengen Area. In such a scenario, OSINT techniques may help to identify and assess this risk in a fairly traditional manner: Journalistic texts published online from that neighboring country may mention the occurrence of the theft. A systematic analysis of online news articles may, thus, allow the identification of a heightened risk of plastic explosives being smuggled across the border, which may result in the decision to change the emphasis of the revelatory function by allocating more resources for corresponding checks. In a collaboration between researchers from the Joint Research Centre of the European Commission, FRONTEX and the University of Helsinki, Atkinson et al. (2010, p. 173) describe a way for “automating the process of extracting structured knowledge from on-line news articles on border-security related events at the EU borders and in related third countries.” Here, the idea of OSINT relates to the risk of so-called illegal migration, but the concept is applicable to public security risks in the same fashion.

Apart from analyzing information that is explicitly and broadly published online to a wide audience, however, OSINT techniques can also be applicable to what has been termed the “deep” or “invisible web” (Warnick et al. 2001), i.e., to parts of the internet that are *not* indexed through common search engines such as Google, Bing, or Baidu.^6^ This could include password-protected databases or websites that are, technically, publicly accessible, but that require knowledge of a specific address. Cloud services often allow sharing photos and other files exclusively with a previously known group of people through this form of protection: A link shared by email allows access only to those who have received it. While, technically, anyone can gain access if they knew where to look, the link, and, thus, the shared information is not “openly available” or “publicly accessible” in the sense designated by OSINT.

The deep web should not be confused with the “darknet.” While the darknet is part of the deep web, i.e., it is not indexed by common search engines, its primary purpose is not to create inaccessibility, but *anonymity*. On a technical level, this is achieved through applications like Tor, which create several layers of encryption. It allows the communication of information from one party to another without either machine knowing the “real” or “unencrypted” network address which could normally expose identifying information. Because anonymity networks like Tor are not indexed, the darknet makes it necessary to know a special address for particular service. Thus, the darknet *can* be used to publish information that is both anonymous as well as unavailable for persons unaware of this address. However, many anonymous darknet services are deliberately *public*. For example, darknet marketplaces depend on being found by those who look for it. Hence, the addresses of such websites are listed in darknet directories or in the surface web.

In the research project TRESSPASS, a “dark web crawler” has been developed that is meant to facilitate situational risk-based border checks (Weydner-Volkmann 2020, p. 26). This technology aims at automatically analyzing known addresses in the Tor network, especially those of known darknet marketplaces, to gather intelligence on specific threat objects being illegally traded and on trends of such trades. In the project, it was researched how such information could be used to differentiate the revelatory function as part of a risk classification of travelers: More resources may be allocated for more intensive checks on travelers that originate from certain regions or on checks for “trending” threat items.

### Ethical Analysis of OSINT for Situational Risk Assessment

In times where much of peoples’ conduct leaves digital traces and where personal interactions and other events in people’s lives happen entirely or at least in part digitally, new forms of shielding mechanisms become increasingly important: Privacy settings in apps and social media platforms allow a certain measure of control of the flow of personal information from one context to another; through encryption and other technologies, we can create “shielded virtual spaces,” where access to digital information is limited to certain persons. Much of what has been termed the “deep web” above is the result of such “digital shielding.”

As part of the revelatory function, digital shielding may be removed during border checks. While physical checks may pose a wholesome negation of such shielding, e.g., by accessing travelers’ personal electronic devices (Brodkin 2019), OSINT by definition seems to be a lot less problematic, since it only deals with *unshielded* information. Furthermore, for situational risk analysis, person-related information is not needed.

However, when we consider darknet OSINT, the picture changes somewhat. A first important factor to consider is how sites are added to the list of addresses to be mined for information. The privacy impact will depend on how well this is limited to sites like marketplaces that are strictly relevant for the *specific security goals* of risk-based border management. Such limitations are especially relevant since, as opposed to most news sites on the clear web, darknet marketplaces are also used to anonymously share or trade *stolen and leaked personal, sensitive or compromising information*, as well as *information that relates to victims of crimes such as sexual abuse*. Indiscriminate mining and analysis may, thus, violate norms of information flow by inadvertently including information that was made public without knowledge and consent of the affected persons and use it as a basis for decision making in border checks. While selective mining could help (e.g., only sites that contain certain keywords are automatically indexed), it seems unlikely that such information could be reliably excluded. Hence, in this case, the privacy impact will depend less on the fact of public availability of the data, but rather on *how well* such processes can prevent inappropriate flows of information.

As mentioned above in Sect. 3, privacy is not the only relevant value concept when we think about the protection of personal data. Another very relevant question is whether such forms of web crawling may chill legitimate usage of the darknet. For example, some investigative journalists have created darknet mailboxes to give whistleblowers the technical means to communicate through reliable means of anonymization^7^—however, it is likely that such exchanges will be protected by additional shielding. Another example may be articles by journalists, bloggers, or NGOs that are meant to be accessible anonymously and hard to censor or “delete” from the web.

Information gathered by a darknet crawler operated within the EU may, in the end, gather and index information from any region in the world. This may pose a problem if there are efforts to analyze this (public) information for clues that may lead to identifying information (e.g., the occurrence of uncommon pseudonyms or used not only in the darknet but also in the clear web). Again, such chilling effects could be reduced or minimized by explicitly limiting the crawling to darknet marketplaces and similar sites. Efforts should be made to prevent the de-anonymization of pseudonyms. While such activities are less relevant for situational risk-based border checks, the mined data and the index may be stolen by third parties or used for purposes other than originally justified and intended (“mission creep”).

Another important value concept to consider was introduced in Sect. 3: non-discrimination. This is because the risk categories created as part of the situational risk-based border checks may include broad risk indicators that accumulate the privacy impact for large groups of travelers, potentially over a long time. For example, if OSINT suggests that there is a higher risk of smuggling explosives among the travelers on flights departing from a specific third country A, a reaction could be to tighten checks for contraband for all of these travelers. Naturally, citizens of country A or their family would be much more affected by this, as they may travel more often from that country into the Schengen Area. If citizens of this country are part of a salient social group, traveler differentiation may result in reproducing structural forms discrimination.

Hence, from an ethical point of view, even where OSINT avoids the collection of private or sensitive data, the relationship between gathering data, creating risk categorizations, and implementing new checks measures cannot be a direct, static one. The discriminatory impact of OSINT in situational risk-based checks will depend on whether the risk categories disproportionately result in more intensive subsequent checks, greater loss in time, etc., for certain societal groups.

This also implies that travelers should be able to learn that they were classified “high risk” due to matching certain situational (non-personal) circumstances and challenge that through the legal process. The more information can be given to the traveler without compromising the security effect of the risk classification, the lower will be the lack of transparency, but also of accountability, as travelers will be enabled to effectively seek legal redress.

### Surface and Deep Web OSINT for Risk Profiling

In this section, I will explore the potential role of OSINT in the context of risk profiling using person-related data. Here, the revelatory function of border checks will vary in intensity based on a risk analysis of “background information” related to specific travelers. This is a highly sensitive political and legal topic. How realistic it is that automated OSINT based risk profiling becomes part of the European border checks regime depends on its conceptual role. After all, as has been shown for the aviation context, the ethical, legal, and societal repercussions can be prohibitively severe (Weydner-Volkmann 2017).

Just like for situational risk assessment, OSINT techniques may be applied to the surface web as well as to the darknet. Surface web OSINT for risk profiling may make use of journalistic texts, e.g., on persons of public interest, and of social media data, but also from other websites such as private blogs or employer websites—as long as the information is publicly accessible. However, this raises the technical challenge of automatically attributing information found online to specific travelers arriving at the border crossing point. Simple name similarities have caused issues in the past in aviation security (Weydner-Volkmann 2018, pp. 169–170). This problem is already difficult on social media as it may be hard to reliably differentiate posts that voice opinions from mere quotations of others. But it becomes fraught with problems in the clear web and even more so in the darknet, where there is often little or misleading context, and where impersonation by malignant third actors becomes another issue.

In the US, starting from around 2016, foreign travelers are, therefore, being asked for their social media account names as part of an online registration (Helmore 2016). At least for automated steps in the analysis, it seems likely that OSINT remains restricted to analyzing the provided account names. However, from a privacy perspective, the technical details of the implementation are crucial: Is it possible for the traveler to decide, at a later point in time, to “curate” information available on them? From a technical point of view, the question is whether (a) the information gathered from the clear web is *stored* for later analysis, and (b) whether further information is used that is outside of the control of the travelers (e.g., older versions of websites or social media profiles at archiving services such as archive.org). We will return to this in Sect. 4.4.

Here, too, it would be a challenge to reliably exclude the processing of information that was not *deliberately* made public by the traveler: Information may have been inadvertently shared to a wider audience than actually intended, or it may have been posted by *others* on social media, e.g., by tagging persons in a shared photo. For the European context, it, therefore, seems unlikely that automated OSINT on social media could become a mandatory part of crossing the border.

In TRESSPASS, another concept of OSINT-based risk profiling was proposed. Here, OSINT was meant to contribute to a trusted traveler program (Weydner-Volkmann 2020, pp. 31–32). In such programs, participating foreign travelers may benefit from relaxed checks at the border crossing point if they voluntarily register in advance and consent to checks of “background information” (including public social media data). Just like in the US, the traveler would identify social media account names as part of the registration process. TRESSPASS’s open web analysis tool was meant to access only public information in the clear web. Information that is redacted (e.g., via a privacy setting in social media), shared only among a set of people (e.g., social media friends), or simply not indexed to prevent unfettered access from anyone was not meant to be used (Weydner-Volkmann 2020, pp. 31–32, 37).

While risk profiling could potentially also be based on OSINT in the darknet, it would be necessary to find other means of attributing information collected from the darknet to specific travelers. Furthermore, it would constitute a particular challenge to automatically assess the validity of this information. Additionally, the legality of using consent to justify profiling becomes highly questionable for information that travelers may not be aware of. In the foreseeable future, it, therefore, seems unrealistic that risk profiling-based OSINT in the darknet will become part of the European border checks regime. In the following section, I will, therefore, focus on concepts involving clear web OSINT, only.

### Ethical Analysis of OSINT for Risk Profiling

One major concern from a privacy perspective relates to the inclusion of *inadvertently* publicized data, i.e., data that were not meant to be made public and, hence, potentially not part of what a traveler had in mind when consenting to a check of “background information” on social media. After all, it is often unclear whether information has deliberately been made public. Default settings on social media platforms may entice people to share much more information more widely than they actually want. Similarly, publicly available information on a traveler may not have been shared by that traveler, but by third parties. Hence, even when consent was obtained, the use of public information on social media can still imply a (further) violation of contextual integrity. This is because the validity of consent from a position of informational disadvantage is highly questionable (Nissenbaum 2004, p. 148): One could argue that one needed to conduct an OSINT analysis on oneself to fully understand the implications and to give *informed* consent in a strong sense.

As outlined above, this issue could be mitigated by enabling travelers to “curate” the checked information. This could be done by limiting OSINT to information that has *currently* been made public *by the traveler*, not by third parties. Contrary, by including archival and third party provided information would exacerbate this issue. Similarly, the systematic compilation of information from different sources (potentially even from data brokering sites) can reveal more information on travelers’ personality than they could foresee when giving consent; such a creation of profiles goes much further than the use of publicly available information. Additionally, the more information on travelers is stored, the higher the privacy impact when this information is breached, leaked, or otherwise misused by criminals or law enforcement. Again, such forms of violations of contextual integrity can be mitigated by avoiding the storage further communication of related information wherever possible.

Another issue with regard to obtaining *informed consent* for social media OSINT relates to the visibility of contacts or “friends.” Those contacts will likely not have consented to being part of the background checks. Hence, contextual integrity would allow for little more than a simple check on whether one or more of the contacts results in a match from a predefined list of persons relevant for risk-based border checks. Apart from these privacy related issues, one should also note that even when social media OSINT is only conducted as part of a *voluntary* procedure, it could still lead to chilling effects. Travelers may refrain from legitimate actions such as voicing political criticism out of fear of no longer being eligible for relaxed checks at the border. However, one of the most prominent concerns regarding risk profiling concerns discriminatory impact. Especially in cases where automated steps of analysis are foreseen, the use of certain indicators can disproportionately affect members of salient social groups. For cases where AI-driven techniques are used for identifying “risk patterns,” this concern is especially endemic, as it often remains opaque *why* certain data patterns result in a specific risk categorization (black box problem)—and in how far members of a vulnerable or disadvantaged group are disproportionately affected.

Since the EU’s legal framework demands humans-in-the-loop [EU 2016c, Preamble (71)], fully automated steps in OSINT data collection and analysis, especially if AI-based steps are foreseen in the future, would need to produce results that are meaningful for decision making. Here, it does not suffice to ensure that risk indicators do not make use of “protected” information such as religious or political beliefs (cf. Sect. 3), but it is also necessary to check for a disproportionate denial of the low-risk status for salient social groups. Hence, irrespective of only making use of public information, the ethical impact of OSINT techniques in border checks will depend on whether their impact leads to a reproduction of structural discrimination. Thus, even when using consent, the ethical impact will depend on transparency and accountability: The more the use of OSINT-based risk profiling can be explained in detail, the more meaningful the possibility of legal redress for being denied a low-risk classification, e.g., on grounds of discrimination. The above does *not* imply that OSINT’s ethical impact is necessarily prohibitive. It *does* show, however, that even when travelers give consent, there are forms of ethical impact that need to be considered—irrespective of the data’s public accessibility.

## Conclusions

The goal of this article was to clarify what OSINT practices could entail in the context of the European border checks regime and to contest the idea that the exclusive use of “open,” “public” data results in an extensive mitigation of the ethical concerns related to risk-based checks on travelers. After sketching out the relevant historical developments toward data-driven, risk-based checks in Sect. 2.1, I have discussed the EU’s border management regime for the Schengen Area and reconstructed its “logic” for border checks in Sect. 2.2. While there is no single coherent purpose that governs the checks, I argued that this logic can be expressed as two main functions: (1) the access and egress control function that operates in a spatial, territorial sense based on (2) the revelatory function, which could include OSINT techniques.

Section 3 then introduced several value concepts that may come into conflict with this revelatory function as part of data-driven, risk-based border checks. Here, the conception of privacy was loosely based on Nissenbaum’s (2004) idea of contextual integrity. Building on Poscher (2017), data protection was conceptualized as a right that deals with situations where data collection poses an abstract danger to values such as privacy, non-discrimination, transparency, and accountability.

At least currently, there is no legal basis that allows and defines the use of OSINT to enable risk-based border checks. Instead, Sects. 4.1 and 4.3 started from two conceptually different variants of risk-based checks, situational risk assessment and risk profiling, to explore its potential role. In Sects. 4.2 and 4.4, I then explored for each of them in how far the use of publicly available data does not preclude fundamental forms of ethical impact.

I hope that the conceptual and evaluative work in this article contributes to two relevant research debates: (1) the controversial use of data-driven risk classification of travelers and (2) the ethical repercussions of OSINT in public security provision. As we have seen, it is not the status of the data that determine the ethical impact of OSINT practices, but the situational details of the implementation.

## Footnotes

1 I thank the anonymous reviewers for their important comments.

2 The plans were part of the smart borders package and consisted in a paid service where travelers would voluntarily provide personal data and undergo a security background check in exchange for facilitated border crossings (Leese 2016, 413). It resulted in the European Travel Information and Authorisation System (ETIAS), which is rather different: When ETIAS will start (likely in 2023), it will require all third-country nationals to provide certain data before arrival so as to perform automated checks against the data bases mentioned above.

3 While Koops, Heopman and Leenes (2013, 677, 683) discuss privacy by design for OSINT tools and underscore the requirement for a clear basis in the law for state authorities, their discussion mainly deals with legal compliancy. I will therefore make use of a different privacy concept in this section.

4 While I use Nissenbaum’s conception of privacy and while I follow Poscher in differentiating privacy from data protection, his conception of data protection allows the inclusion of fundamental values *other than privacy* that have also been part of the longstanding privacy debate (e.g. Rachels 1975).

5 EU Horizon 2020 project “robusT Risk basEd Screening and alert System for PASSengers and luggage” (TRESSPASS), Grant agreement ID: 787,120, https://doi.org/10.3030/787120.

6 Studies on the size of the deep web vary greatly in their assessments. Warnick et al. (2001) cite a whitepaper that estimates its size as “500 times that of the surface web.”.

7 For example, the Tor address http://sq4lecqyx4izcpkp.onion/ leads to the mailbox of “heise Investigativ”.
